# Plasma chemistry in nesting leatherback sea turtles (*Dermochelys coriacea*) from Florida: Understanding the importance of sample hemolysis effects on blood analytes

**DOI:** 10.1371/journal.pone.0222426

**Published:** 2019-09-10

**Authors:** Nicole I. Stacy, Ryan M. Chabot, Charles J. Innis, Carolyn Cray, Katelyn M. Fraser, Kimberly S. Rigano, Justin R. Perrault

**Affiliations:** 1 Aquatic, Amphibian, and Reptile Pathology Program, Department of Comparative, Diagnostic, and Population Medicine, College of Veterinary Medicine, University of Florida, Gainesville, Florida, United States of America; 2 Division of Comparative Pathology, Department of Pathology & Laboratory Medicine, University of Miami Miller School of Medicine, Miami, Florida, United States of America; 3 Inwater Research Group, Inc., Jensen Beach, Florida, United States of America; 4 New England Aquarium, Boston, Massachusetts, United States of America; 5 Loggerhead Marinelife Center, Juno Beach, Florida, United States of America; University of Minnesota, UNITED STATES

## Abstract

Plasma chemistry is widely used in diagnostic and research settings in sea turtles. However, plasma discolorations such as hemolysis are often not considered in data interpretation. The objectives of this study were to (1) evaluate the effects of moderate hemolysis on plasma electrolytes, minerals, and proteins using dry chemistry analysis (DCA) and protein electrophoresis from nesting leatherback sea turtles (*Dermochelys coriacea*) from Florida and to (2) establish blood analyte reference intervals. Twenty-six plasma samples with absence of hemolysis were selected and sub-divided into one non-hemolytic aliquot and an aliquot that was experimentally manipulated to mimic moderate hemolysis. Plasma samples were analyzed for hemoglobin using a handheld photometer; sodium, potassium, chloride, magnesium, calcium, phosphorus, and total protein using DCA; and protein electrophoresis. Packed cell volume and hemoglobin were measured in corresponding whole blood samples. Reference intervals were established. All analytes except calcium and pre-albumin were significantly higher and the calcium:phosphorus and albumin:globulin ratios were significantly lower in hemolytic plasma compared to non-hemolytic plasma. Alpha_2_-globulins and potassium were the analytes most impacted by hemolysis, averaging 3.3- and 2.0-fold higher in hemolyzed samples, respectively, indicating that (1) hemoglobin migrates into the alpha_2_-globulin region in this species and (2) notable intracellular potassium is released into plasma with hemolysis. Attempted conversion factors for compensation of hemolysis were considered inaccurate for 4 of 16 analytes due to non-significant regression lines. We also report that PCV provides an estimate of hemoglobin (g/L) using the formula: (2.59 × PCV) + 24.59. Given the spurious effects of hemolysis, the degree of this artifact should be reported with biochemistry data, and samples with moderate to severe hemolysis should be excluded from datasets when interpreting electrolyte, mineral, and protein results. This will ensure accurate data interpretation for individual turtles in rehabilitation or research settings and population-level data relevant to conservation-focused projects.

## Introduction

Plasma chemistry analysis is well-established in laboratory diagnostics for individual sea turtles during rehabilitation, investigations of physiological variations such as nesting and other life-stage class differences, and in understanding population-level effects from diseases and various stressors such as cold-stunning, forced submergence, or oil spills [1]. This use of blood chemistry in diagnostic and research settings is similar to any other species, from invertebrate hemolymph to plasma or serum in terrestrial mammals, birds, and marine mammals. Detailed guidelines about sample quality for chemistry analysis in human and veterinary clinical pathology have been reported in textbooks and peer-reviewed publications, with emphasis on pre-analytical factors including hemolysis, lipemia, and icterus [2,3].

Hemolysis is defined as red discoloration of plasma and is consistent with the presence of free hemoglobin. It occurs after rupture of erythrocytes (red blood cells; RBC) with consequent release of cytoplasmic constituents into the plasma, resulting in variably red discolored plasma after removal of cellular components after centrifugation. Sample hemolysis can occur *in vivo* as a pathologic condition associated with hemolytic anemia or *in vitro* during the pre-analytical phase of sample handling and processing. *In vivo* hemolysis is typically accompanied by a decreased packed cell volume (PCV) and can result from various clinically important conditions resulting in RBC destruction in veterinary species, including infections (e.g., bacterial, viral, hemoparasitic), RBC metabolic defects (e.g., oxidative damage), intravascular RBC fragmentation, and physical, immunologic, or chemical causes. Although the PCV is expected to decrease with *in vivo* hemolysis, the decrease may not initially be great enough to diagnose the severity of the patient’s condition. In addition, concurrent dehydration may mask the degree of anemia. Therefore, a low normal or normal PCV with concurrent hemolysis does not exclude the possibility of a hemolytic condition. Additional diagnostics (e.g., urinalysis to identify hemoglobinuria) are indicated whenever hemolysis is observed in any sample.

*In vitro* sample hemolysis often occurs during or after blood sample collection and processing. Common causes for artifactual hemolysis include increased negative syringe pressure (e.g., difficult blood withdrawal), direct contact of the blood tube with ice during transport, exposure to warm temperatures, delayed removal of serum or plasma from RBC, presence of lipemia, and, specifically in chelonian and some amphibian, fish, and avian species, blood anticoagulation into ethylenediaminetetraacetic acid (EDTA) [4,5]. Understanding the effects of hemolysis is critical when interpreting blood chemistry data. The mechanisms of hemolysis interference include (1) release of RBC constituents into the sample, (2) sample dilution, which is relevant for selected analytes with severe hemolysis (e.g., albumin, glucose, sodium), (3) chemical interference by directly or indirectly affecting various biochemical reactions in assays and in spectrophotometrical analysis, and (4) by affecting reactions with antigens and/or antibodies in immunochemistry assays [3]. The effect of the release of RBC constituents into plasma is well understood in humans and to a lesser extent in domestic species. Studies in reptiles are few [6,7], and some studies exist for other non-mammalian species such as fish and birds [8–10]. One important intracellular RBC constituent is potassium, the concentration of which is 40 times higher in human RBC than in plasma [3]. Intra-RBC potassium concentrations are reportedly high in horses, pigs, and cattle, but typically low in dogs except for Akita and other Japanese breed dogs [5]. It can be concluded that the amount of potassium release from RBC is species- and even breed-dependent. Given the narrow range for plasma potassium concentration, and clinical implications for its interpretation, spurious increases (i.e., pseudohyperkalemia secondary to hemolysis) can result in an inaccurate diagnosis [3,5].

Sea turtle species world-wide are vulnerable or endangered. The leatherback sea turtle (*Dermochelys coriacea*) is listed as vulnerable internationally [11]. Blood analysis in this and other sea turtle species is an integral part of studies on biology and physiology, response to stressors, and disease [1, 12–20]. Sampling blood from sea turtles in the field (e.g., nesting turtles on the beach, in-water capture-release studies) can pose tremendous challenges in ensuring optimal sample quality, such as avoiding hemolysis. In addition, these samples are often unique and precious due to the logistical efforts in successfully acquiring blood from free-ranging wildlife, acquisition of permits on state, federal and/or international levels, funding limitations in wildlife research, and the near impossibility of collecting additional samples from the same individual. There is often only one opportunity to obtain a high-quality sample. Thus, all efforts need to be made to ensure optimal sample quality and to avoid any artifact from sample collection, handling, and processing. Since the effects of hemolysis in sea turtles have not been studied, RBC chemistry is unknown, and information about presence or absence of hemolysis is often not included in the published literature, there is a need to understand the effects of hemolysis on chemistry data in sea turtles. The objectives of this study were to (1) evaluate the effects of moderate hemolysis on plasma electrolytes, minerals, and proteins using dry chemistry analysis (DCA) and protein electrophoresis from nesting leatherback sea turtles (*Dermochelys coriacea*) from Florida and to (2) establish blood analyte reference intervals. The hypothesis was that hemolysis would have several effects on chemistry analytes resulting in potential biases in interpretation of data.

## Materials and methods

### Ethics statement

Our study was carried out in accordance with Florida Fish and Wildlife Conservation Commission Marine Turtle Permits #18–205 and #18–021. University of Florida’s Institutional Animal Care and Use Committee (IACUC) approved this study (#201706823).

### Sample collection and processing

From April–June, 2018, sample collection from nesting leatherbacks was conducted on Juno Beach and Jupiter Beach, Florida USA (26.836449°N, –80.041231°W to 26.943210°N, –80.071726°W). Turtles were sampled during egg deposition after the turtles had entered their nesting fixed action pattern. After disinfecting the venipuncture site (femoral rete system) with 70% isopropyl alcohol swabs, up to 10 mLs blood were collected using Vacuette^®^ 20 G x 1½” needles (Vacuette, Greiner Bio-One, Kremsmünster, Austria) fitted into 10 mL BD sodium heparin glass Vacutainer^®^ tubes (Becton-Dickinson and Co., Franklin Lakes, New Jersey USA). Blood was kept on ice in the field immediately after collection and was processed within 6–8 hours. PCV from whole blood was determined using capillary tubes (Fisher HealthCare, Houston, Texas, USA) centrifuged for 8 minutes at 4,200 g (5,000 rpm) using an LW Scientific C5 centrifuge (Lawrenceville, Georgia, USA) with microhematocrit tube inserts. A subset of whole blood was frozen at –80°C and the remaining blood was centrifuged at 4250 *g* (3,500) rpms for 10 minutes. Plasma was harvested and immediately stored at –80°C and analyzed within a maximum of 90 days.

### Plasma preparation

**Fig 1:**
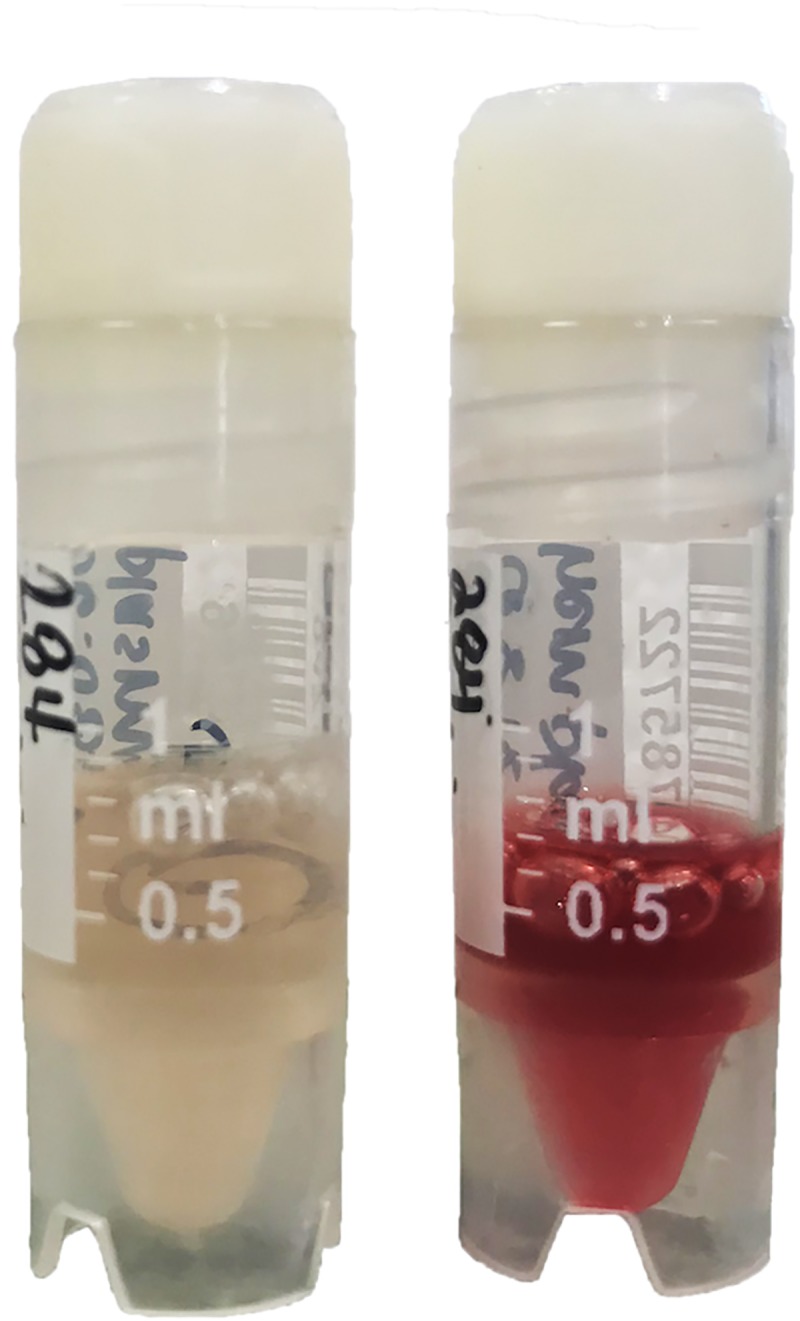
Paired non-hemolytic plasma from leatherback sea turtles (*Dermochelys coriacea*) (i.e., score = 0) is shown on the left, while hemolyzed plasma (i.e., score = 2+) is shown on the right.

Plasma samples with absence of hemolysis were selected for use in this study and were sub-divided into two aliquots: one subsample was kept native (i.e., non-hemolytic), while hemolysis artifact was introduced to the other subsample using thawed whole blood from the same individual turtle. Freezing results in disruption of cellular membranes; thus, all cells in whole blood samples are expected to be ruptured after freezing at ultrafreezer temperature (i.e., –80°C). This was further confirmed by the absence of visible cell pellets after centrifuging thawed whole blood samples (4200 *g* for 10 minutes). Based on routine laboratory procedures, plasma hemolysis is typically scored on a scale from 0–3+ by visual evaluation, with 0 indicating a non-hemolytic sample (i.e., clear plasma color), and 1+, 2+, and 3+ indicating mild, moderate, and marked hemolysis, respectively [1]. In this study, we compared non-hemolytic (i.e., 0 scale) and moderately (i.e., 2+ scale) hemolytic samples. To prepare a 2+ hemolytic sample, 40 μL of thawed whole blood was added to 400 μL of the second subsample of the non-hemolytic plasma of the same turtle, resulting in “cherry-red” color (Fig 1).

### Hemoglobin analysis

Hemoglobin in frozen-thawed whole blood, non-hemolytic plasma, and moderately hemolytic plasma was analyzed using a HemoCue^®^ Hb 201^+^ photometer (HemoCue^®^, Inc., Lake Forest, California USA) with HemoCue^®^ Hb 201 microcuvettes. This hemoglobin analytical methodology has been validated for use in birds and hawksbill sea turtles [21–23] and uses a modified azidemethemoglobin reaction. To evaluate for potential interference of lysed nuclear chromatin with hemoglobin concentrations by spectrophotometrical analysis of the HemoCue^®^ Hb 201, frozen-thawed whole blood was (1) vortexed only and not centrifuged and (2) vortexed and centrifuged to remove any potential nuclear chromatin remnants. Since there was no significant difference using a paired t-test (t[18] = 0.16; *P* = 0.87) between hemoglobin in vortexed blood (mean ± SD: 115.7 ± 14.0 g/L) and hemoglobin in centrifuged blood (using supernatant to analyze for hemoglobin: 114.7 ± 13.9), the additional centrifugation step was omitted. This is similar to the methodology by Velguth *et al*. [23] during which this centrifugation step was not included. To quantify hemoglobin, ~20 μL of thawed whole blood, non-hemolytic plasma, and hemolytic plasma were drawn into heparinized capillary tubes. The blood was subsequently transferred into the HemoCue^®^ microcuvettes by capillary action and placed into the HemoCue^®^ photometer for analysis. The measuring range of this machinery is from 0–256 g/L.

### Electrolytes, minerals, and plasma protein analyses

Paired non-hemolytic and hemolytic (2+) plasma samples were shipped overnight on dry ice to the University of Miami Avian & Wildlife Laboratory (Miami, Florida USA). Electrolytes (sodium, potassium, chloride), minerals (magnesium, calcium, phosphorus), and total protein (by biuret method) were analyzed using the dry slide chemistry analyzer Ortho 250XR (Ortho Clinical Diagnostics, Rochester, New York USA). Protein fractions were quantified using gel electrophoresis and laser densitometry using previously reported methodology [24–25]. Each protein fraction (pre-albumin, albumin, alpha_1_-globulins, alpha_2_-globulins, beta-globulins, gamma-globulins) was calculated using the percentage of the fraction multiplied by the total protein concentration.

### Statistical analysis

Statistical analyses were performed using IBM SPSS Statistics 25 software (SPSS, Inc. Chicago, Illinois USA) and R [26]. Differences between means of analytes in non-hemolytic and hemolytic plasma were determined using a paired-samples t-test. Any outliers (i.e., >2.2 interquartile ranges higher or lower than the first or third quartile) [27] in the differences between the two groups were removed from the analysis to meet the assumptions of this test. Normality of the differences between the two groups was assessed using the Shapiro-Wilk test. All *P* values for the paired-samples t-test were adjusted using the Bonferroni correction (P = 0.05/17 analytes = 0.0029). Linear least-squares regressions were used to (1) establish a conversion equation between PCV and hemoglobin concentrations in whole blood, (2) to determine if any blood analytes significantly decreased during nesting season (independent variable = date of nesting encounter), and (3) determine relationships between hemoglobin in whole blood and hemolyzed plasma with hemolytic plasma electrolytes, minerals, and proteins. Normality of the regression residuals was assessed using the Shapiro-Wilk statistic. Normality of the regression residuals was assessed using the Shapiro-Wilk statistic.

Reference intervals for all blood analytes were calculated using MedCalc (MedCalc Software v.18.5, Ostend, Belgium) following the American Society of Veterinary Clinical Pathology reference interval guidelines, using parametric methods for sample sizes ≥20 but ≤40 [28]. Normality was assessed using the D’Agostino-Pearson test, while outliers were detected using the Reed test. Box-Cox transformation was employed when necessary.

### Analyte conversion equations

We constructed conversion equations for each analyte measured in hemolyzed and non-hemolyzed plasma samples using standardized major-axis (SMA) regression models with the package “smatr” in R [26,29]. These models produce quantitative estimates of the relationships between data collected from hemolyzed and non-hemolyzed plasma and provide an indication of the extent to which hemolysis may alter blood chemistry metrics for each analyte. Models constructed using SMA regression are superior to those creating using ordinary least-squares (OLS) regression for this task. Equations generated using SMA regression can be used to exchange values bidirectionally, while those from OLS regression should only be used to predict a dependent variable from values of an independent variable unidirectionally because of caveats surrounding error structure and slope estimates [30–32].

## Results

Whole blood and non-hemolytic and hemolytic plasma samples were collected from 23 nesting leatherback turtles for a total of 26 samples (3 turtles were sampled twice at two different time points during the nesting season).

**Fig 2:**
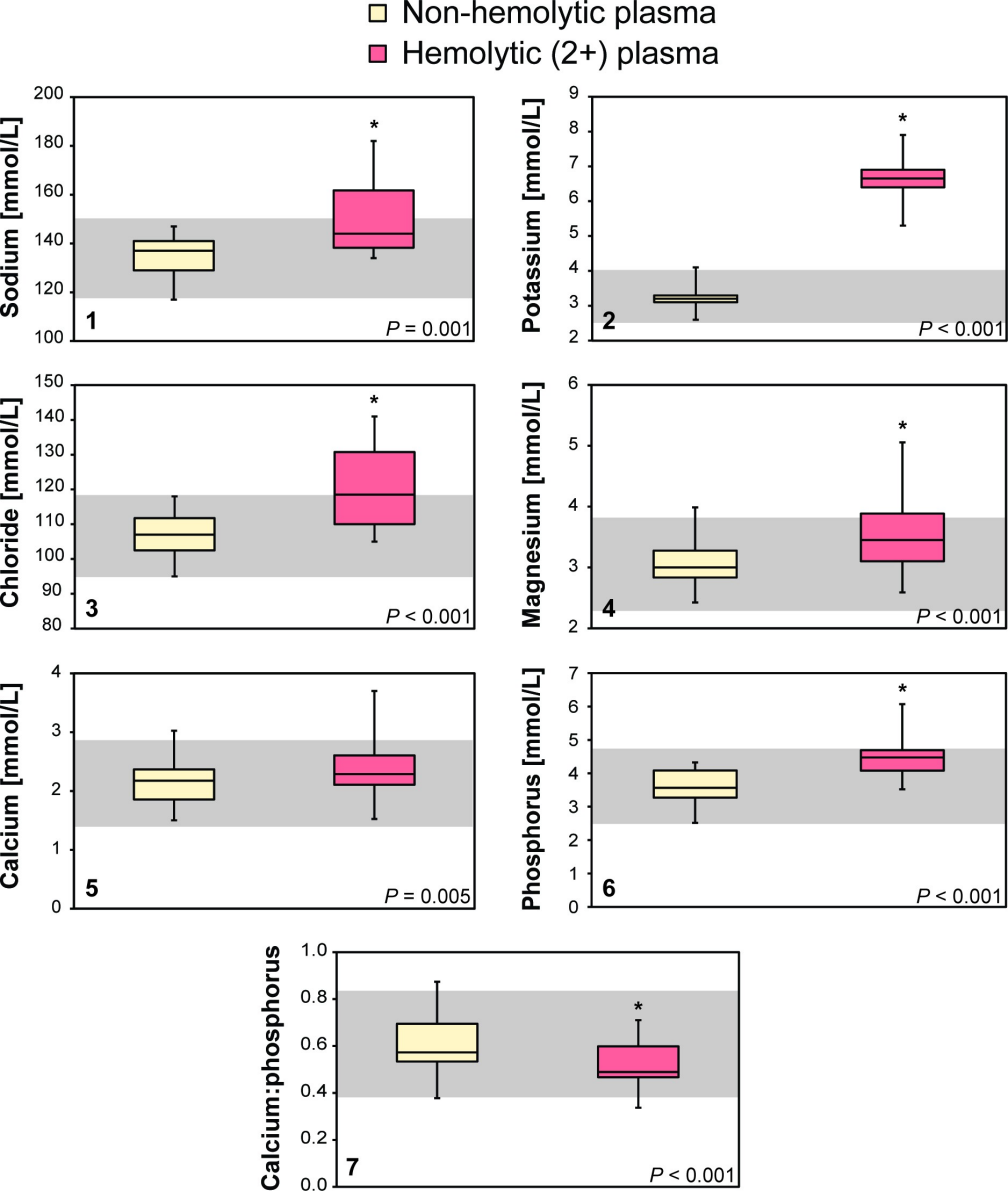
Comparison of electrolytes (1: sodium; 2: potassium; 3: chloride) and minerals (4: magnesium; 5: calcium; 6: phosphorus) in non-hemolytic and hemolytic plasma from leatherback sea turtles (*Dermochelys coriacea*). The line represents the median, the boxes represent the first and third quartiles, while the whiskers represent the range. * indicates a significant difference between the two samples as determined by a paired-samples t-test (*P* < 0.0033). Data is presented in SI units.

**Fig 3:**
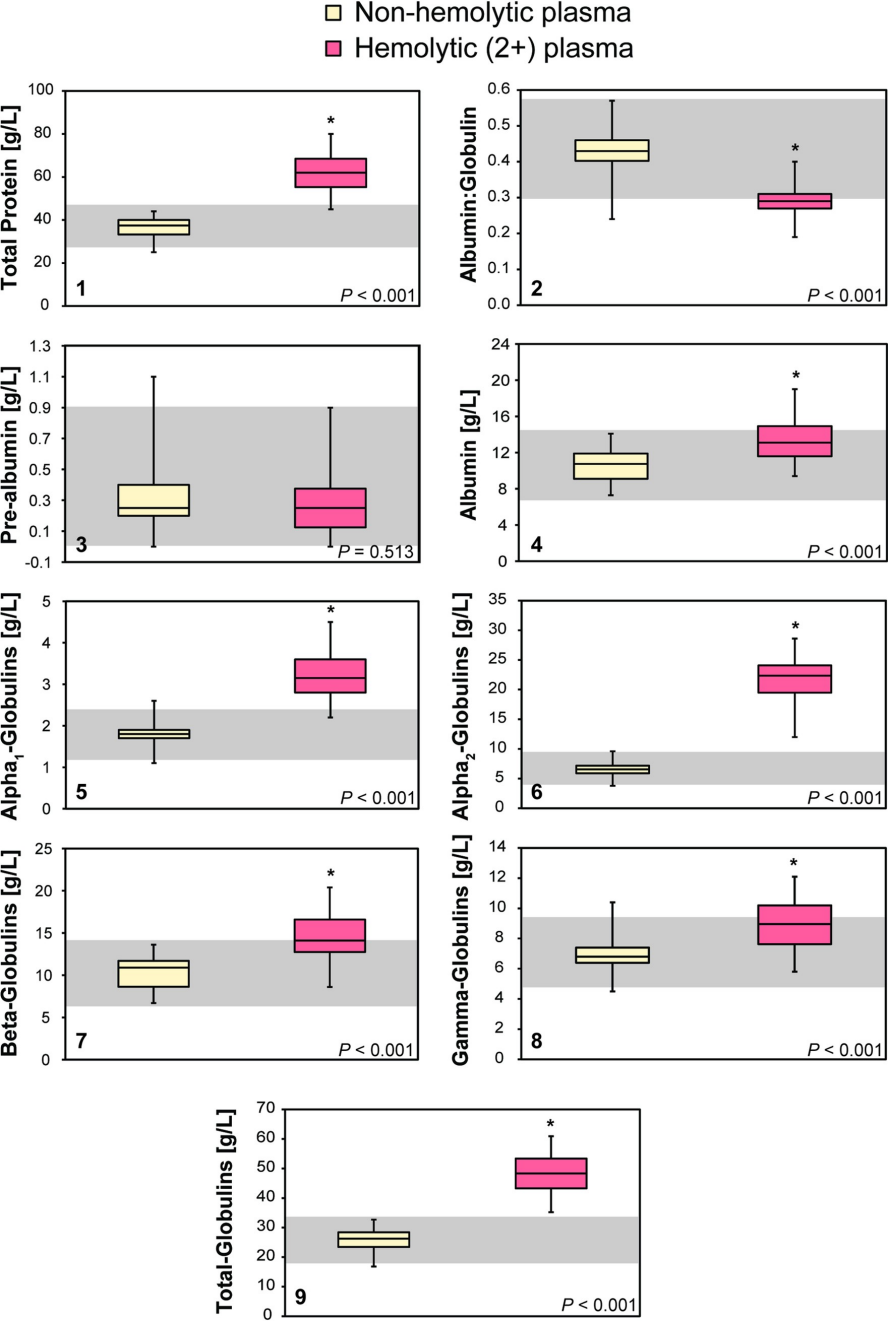
Comparison of proteins (1: total protein; 2: albumin:globulin ratio; 3: pre-albumin; 4: albumin; 5: alpha_1_-globulins; 6: alpha_2_-globulins; 7: beta-globulins; 8: gamma-globulins) in non-hemolytic and hemolytic plasma from leatherback sea turtles (*Dermochelys coriacea*). The line represents the median, the boxes represent the first and third quartiles, while the whiskers represent the range. * indicates a significant difference between the two samples as determined by a paired-samples t-test (*P* < 0.0033). Data is presented in SI units.

**Fig 4:**
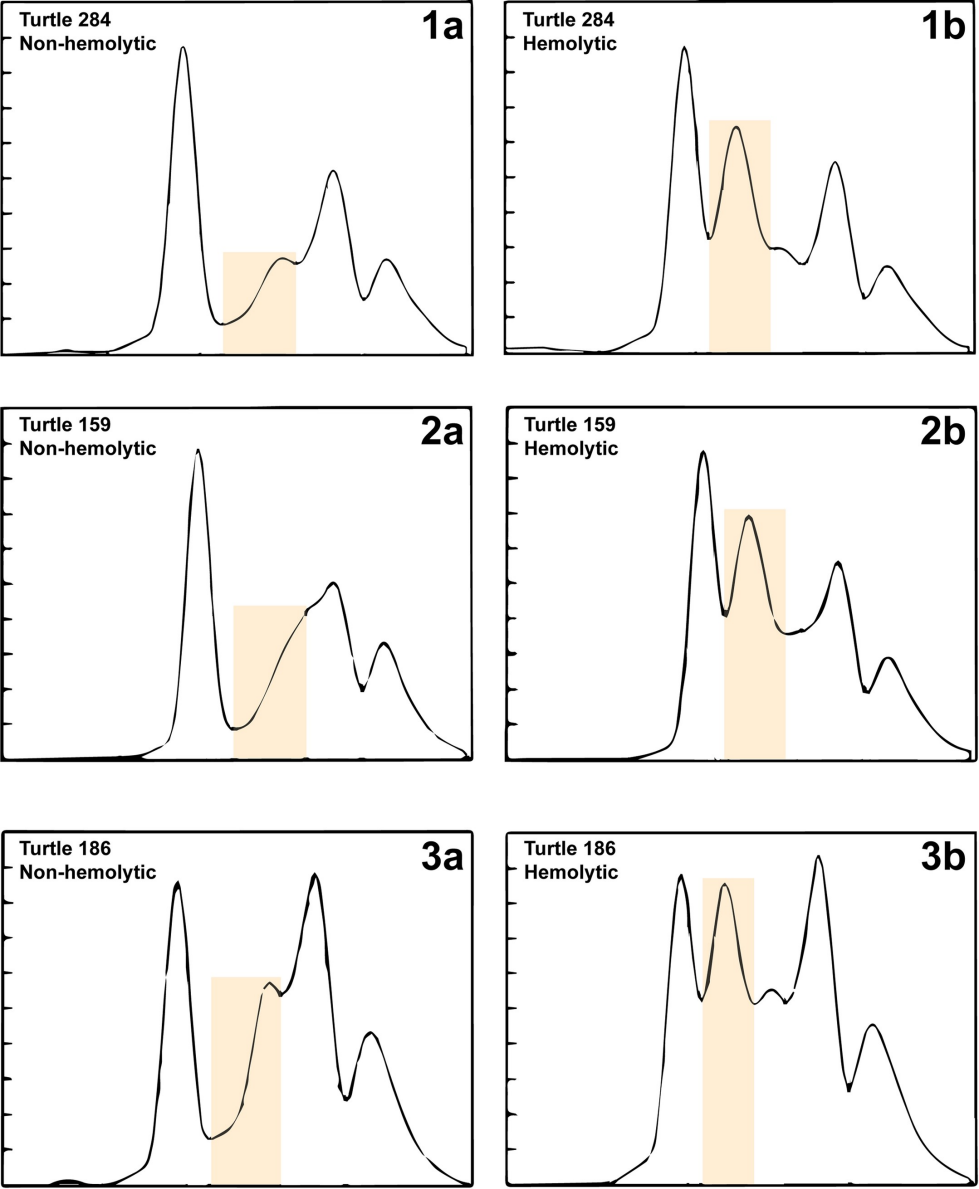
**Electrophoretograms of three individual nesting leatherback sea turtles (*Dermochelys coriacea*) without (left column) and with (right column) hemolysis showing a prominent alpha**_**2**_**-globulin fractions as highlighted in orange in hemolyzed samples.** The orange boxes serve to highlight the alpha_2_-globulin fraction.

Hemoglobin concentrations in whole blood were significantly higher (t[25] = 35.67; *P* < 0.001) than hemoglobin concentrations in hemolytic plasma (Table 1). Hemoglobin concentrations in all non-hemolytic plasma samples were equal to 0 g/L. Sodium (t[25] = –3.76; *P* = 0.001), potassium (t[25] = –25.55; *P* < 0.001), chloride (t[25] = –4.32; *P* < 0.001), magnesium (t[25] = –4.23; *P* < 0.001), phosphorus (t[25] = –6.97; *P* < 0.001), total protein (t[25] = –16.49; *P* < 0.001), albumin (t[25] = –6.56; *P* < 0.001), alpha_1_-globulins (t[25] = –10.13; *P* < 0.001), alpha_2_-globulins (t[25] = –27.73; *P* < 0.001), beta-globulins (t[25] = –8.34; *P* < 0.001), gamma-globulins (t[25] = –7.44; *P* < 0.001), and total globulins (t[25] = –19.66; *P* < 0.001) were significantly higher in hemolytic plasma in comparison to non-hemolytic plasma (Table 1; Figs 2 and 3). The calcium:phosphorus ratio (t[25] = 11.86; *P* < 0.001) and albumin:globulin ratio (t[25] = 18.77; *P* < 0.001) were significantly lower in hemolytic plasma in comparison to non-hemolytic plasma (Table 1; Fig 3). Three electrophoretograms without and with hemolysis are presented in Fig 4.

**Table 1:** Hemoglobin, electrolytes, minerals, and plasma protein fractions by plasma protein electrophoresis in non-hemolytic and hemolytic plasma of 26 nesting leatherback sea turtles (*Dermochelys coriacea*). Reference intervals are indicated for non-hemolyzed plasma. Parametric methods for sample sizes ≥20 but ≤40 were used to calculate reference intervals after Friedrichs et al. 2012. Normality was assessed using the D’Agostino-Pearson test, while outliers were detected using the Reed test. Whole blood hemoglobin results also shown. NA = not applicable. Data is presented in SI units.

Analyte	Hemolysis	Mean	SE	SD	Median	Min	Max	Lower limit (90% CI)	Upper limit (90% CI)
Whole blood hemoglobin [g/L]	NA	117	3	16	115	94	165	93 (89–98)^a^	159 (142–184)^a^
Packed cell volume [%]	NA	36	1	1	36	29	46	27 (25–30)	44 (42–47)
Plasma hemoglobin (g/L)	0	0	0	0	0	0	0	0 (0)	0 (0)
	2+	8.5	0.4	2.2	8.0	2.0	13.0		
Sodium [mmol/L]	0	134.6	1.5	7.9	137.0	117.0	147.0	119 (115–124)	150 (146–154)
	2+	150.1	2.8	14.1	144.0	134.0	182.0		
Potassium [mmol/L]	0	3.2	0.1	0.3	3.2	2.6	4.1	2.7 (2.6–2.8)^a^	4.0 (3.7–4.3)^a^
	2+	6.6	0.1	0.6	6.7	5.3	7.9		
Chloride [mmol/L]	0	107.0	1.2	6.2	107.0	95.0	118.0	95 (91–98)	119 (116–123)
	2+	120.8	2.4	12.0	118.5	105.0	141.0		
Magnesium [mmol/L]	0	3.1	0.1	0.4	3.0	2.4	4.0	2.3 (2.1–2.5)	3.8 (3.6–4.1)
	2+	3.6	0.1	0.6	3.5	2.6	5.1		
Calcium [mmol/L]	0	2.2	0.1	0.4	2.2	1.5	3.0	1.4 (1.2–1.6)	2.9 (2.7–3.1)
	2+	2.4	0.1	0.4	2.3	1.5	3.7		
Phosphorus [mmol/L]	0	3.6	0.1	0.5	3.6	2.5	4.3	2.5 (2.2–2.9)	4.7 (4.4–5.0)
	2+	4.5	0.1	0.6	4.5	3.5	6.1		
Calcium:phosphorus	0	0.61	0.02	0.12	0.57	0.38	0.87	0.38 (0.31–0.44)	0.83 (0.77–0.90)
	2+	0.52	0.02	0.09	0.49	0.34	0.71		
Total Protein [g/L]	0	37	1	5	38	25	44	27 (24–30)	46 (44–49)
	2+	62	2	9	62	45	80		
Albumin:Globulin	0	0.43	0.01	0.07	0.43	0.24	0.57	0.30 (0.26–0.34)	0.57 (0.53–0.61)
	2+	0.29	0.01	0.04	0.29	0.19	0.40		
Pre-albumin [g/L]	0	0.31	0.05	0.25	0.25	0	1.10	0 (0–0.1)^a^	0.9 (0.7–1.2)^a^
	2+	0.29	0.04	0.22	0.25	0	0.90		
Albumin [g/L]	0	10.62	0.37	1.90	10.75	7.30	14.10	6.9 (5.8–8.0)	14.4 (13.3–15.4)
	2+	13.52	0.50	2.57	13.10	9.40	19.00		
Alpha_1_-globulins [g/L]	0	1.80	0.06	0.30	1.80	1.10	2.60	1.2 (1.0–1.4)	2.4 (2.2–2.6)
	2+	3.23	0.12	0.62	3.15	2.20	4.50		
Alpha_2_-globulins [g/L]	0	6.59	0.26	1.35	6.55	3.80	9.60	4.0 (3.2–4.7)	9.2 (8.5–10.0)
	2+	21.58	0.67	3.42	22.35	12.00	28.60		
Beta-globulins [g/L]	0	10.39	0.39	2.00	10.90	6.70	13.60	6.5 (5.4–7.6)	14.3 (13.2–15.4)
	2+	14.43	0.60	3.04	14.10	8.60	20.40		
Gamma-globulins [g/L]	0	6.84	0.23	1.16	6.80	4.50	10.40	4.8 (4.3–5.3)^a^	9.3 (8.5–10.1)^a^
	2+	8.98	0.33	1.67	8.95	5.80	12.10		
Total globulins [g/L]	0	25.62	0.76	3.87	26.25	16.80	32.70	18.0 (15.9–20.2)	33.2 (31.0–35.4)
	2+	48.22	1.43	7.27	48.35	35.20	60.90		

^a^ Reference intervals were calculated using Box-Cox transformations, as data were non-normal.

**Fig 5:**
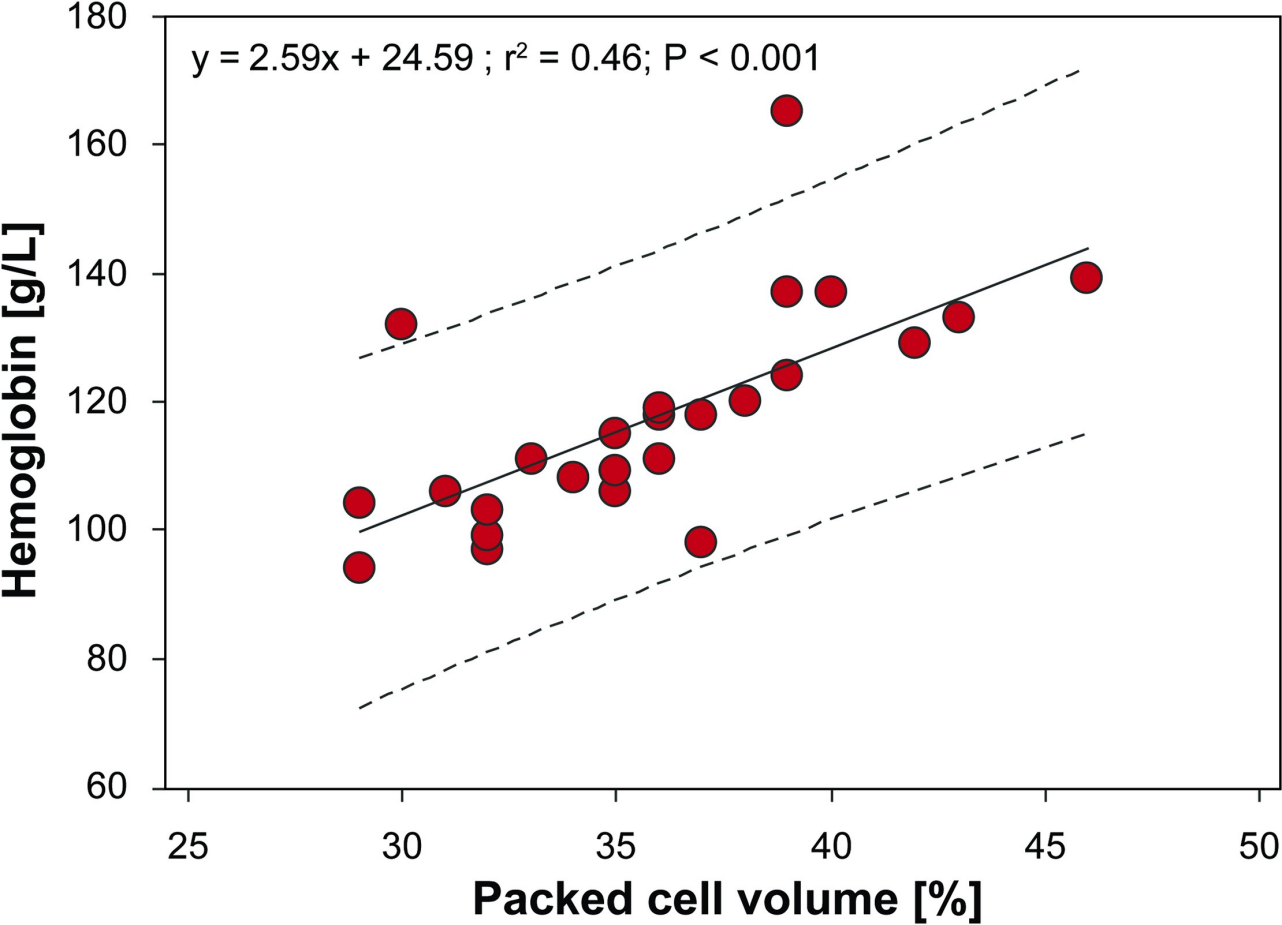
Least-squares linear regression between whole blood hemoglobin concentrations and packed cell volume in nesting leatherback sea turtles. The dashed lines represent the 95% confidence interval of the slope.

Linear regression analysis revealed a significant relationship (*P* < 0.001) between hemoglobin concentrations from thawed whole blood and PCV (Fig 5), which can be described as:

Hemoglobin[g/L]=(2.59×PCV)+24.59

Using this equation, the mean ± standard deviation coefficient of variation between our real hemoglobin values (using the Hemocue^®^ photometer) and the calculated values (using the equation above) was 4.4% ± 5.2%, indicating that hemoglobin concentrations can be accurately predicted by PCV measurement in nesting leatherback turtles. A simplified factor of the hemoglobin and PCV correlation is reflected in the following: hemoglobin [g/L]/3.25 = PCV (SI unit). Hence, the hemoglobin concentration is about 3 times the PCV (PCV x 3.25) using the SI unit (g/L) or a third of the PCV (PCV x 0.325) using the conventional unit (g/dL).

Whole blood hemoglobin concentrations were also positively correlated with a number of analytes in the hemolytic plasma, including potassium (*r*^*2*^ = 0.20; *P* = 0.024), total protein (*r*^*2*^ = 0.20; *P* = 0.021), albumin (*r*^*2*^ = 0.27; *P* = 0.007), and alpha_2_-globulins (*r*^*2*^ = 0.29; *P* = 0.004) (Table 2). Lastly, hemoglobin concentrations in hemolytic plasma were positively correlated with potassium (*r*^*2*^ = 0.24; *P* = 0.010), total protein (*r*^*2*^ = 0.21; *P* = 0.018), alpha_2_-globulins (*r*^*2*^ = 0.58; *P* < 0.017), and total globulins (*r*^*2*^ = 0.21; *P* = 0.020) in hemolytic plasma (Table 2).

**Table 2:** Significant results of least-squares linear regressions comparing hemoglobin (Hbg) in whole blood (WB) and hemolyzed plasma (HP) to plasma electrolytes, minerals, and proteins. Equations for the regression lines are provided, in addition to the confidence intervals (CI) of the slopes of the regression lines and the y-intercept. “2+” indicates a comparison with blood health analytes in hemolyzed plasma.

Comparisons	Regression equation	Slope 95% CI	Intercept 95% CI	*r*^*2*^	*P*	N
WB Hbg v 2+ potassium	y = 0.02x + 4.73	(3.09, 6.37)	(0.002, 0.03)	0.20	0.024	26
WB Hbg v 2+ total protein	y = 0.25x + 32.79	(8.13, 57.45)	(0.04, 0.46)	0.20	0.021	26
WB Hbg v 2+ albumin	y = 0.08x + 4.03	(–2.65, 10.71)	(0.03, 0.14)	0.27	0.007	26
WB Hbg v 2+ alpha_2_-globulins	y = 0.11x + 8.38	(–0.37, 17.14)	(0.04, 0.19)	0.29	0.004	26
HP Hbg v 2+ potassium	y = 0.13x + 5.50	(4.63, 6.36)	(0.03, 0.23)	0.24	0.010	26
HP Hbg v 2+ total protein	y = 1.88x + 46.10	(32.73, 59.46)	(0.35, 3.40)	0.21	0.018	26
HP Hbg v 2+ alpha_2_-globulins	y = 1.16x + 11.71	(8.01, 15.40)	(0.74, 1.58)	0.58	<0.001	26
HP Hbg v 2+ total globulins	y = 1.48x + 35.65	(24.91, 46.40)	(0.26, 2.70)	0.21	0.020	26

SMA regression models revealed significant correlations between hemolyzed and non-hemolyzed values of sodium, chloride, magnesium, calcium, calcium:phosphorus ratio, total protein, albumin, alpha_2_-globulins, beta-globulins, gamma-globulins, total globulins, and albumin:globulin ratio (*P* < 0.05). No significant relationships between values of potassium, phosphorous, pre-albumin, and alpha_1_-globulins (*P* > 0.05) were observed. We used these model results to create conversion equations and report how well the models fit the data using *r*^*2*^ values (Table 3). Model fit was relatively low (mean *r*^*2*^ among all analyte models = 0.30 ± 0.22, range = <0.01–0.75) considering hemolyzed and non-hemolyzed values were derived from the same blood samples.

**Table 3:** Significant (*P* < 0.05) conversion equations between analyte values in non-hemolytic and hemolytic plasma of nesting leatherback sea turtles (*Dermochelys coriacea*) generated using standardized major-axis (SMA) regression. Values can be converted birectionally. Slope and intercept 95% confidence intervals (CI) identify variability in model coefficients. R^2^ values indicate how well models fit the data; values close to zero indicate low fit, while those close to one indicate higher fit. Analytes not included in this Table showed non-significant equations and are not reported here.

Variable	Conversion Equation	Slope 95% CI	Intercept 95% CI	R^2^	p-value
Sodium (mmol/L)	Sodium_Hemolyzed_ = –1.80*Sodium_Non-hemolyzed_ + 391.95	(–2.28, –1.42)	(334.28, 449.63)	0.681	<0.001
Calcium (mmol/L)	Calcium_Hemolyzed_ = 1.16*Calcium_Non-hemolyzed_− 0.15	(0.86, 1.57)	(–0.93, 0.63)	0.473	<0.001
Calcium:phosphorus (Ca:P) ratio	Ca:P_Hemolyzed_ = 0.76*Ca:P_Non-hemolyzed_ + 0.07	(0.69, 0.83)	(0.02, 0.11)	0.951	<0.001
Albumin:globulin (A:G) ratio	A:G_Hemolyzed_ = 0.61*A:G_Non-hemolyzed_ + 0.03	(0.49, 0.75)	(–0.03, 0.08)	0.745	<0.001
Albumin (g/L)	Albumin_Hemolyzed_ = 1.35*Albumin_Non-hemolyzed_− 0.82	(0.95, 1.92)	(–6.07, 4.43)	0.275	0.006
Alpha_2_-globulins (g/L)	Alpha_2-Hemolyzed_ = 2.54*Alpha-2_Non-hemolyzed_ + 4.84	(1.85, 3.49)	(–0.70, 10.38)	0.412	<0.001
Gamma-globulins (g/L)	Gamma_Hemolyzed_ = 1.44*Gamma_Non-hemolyzed_− 0.87	(1.01, 2.05)	(–4.50, 2.76)	0.263	0.007
Total globulins (g/L)	Globulins_Hemolyzed_ = 1.88*Globulins_Non-hemolyzed_ + 0.07	(1.35, 2.62)	(–16.48, 16.63)	0.352	0.001

Blood analyte data from non-hemolyzed plasma samples are reported as reference intervals in Table 1. Potassium, albumin:globulin (A:G) ratio, and albumin decreased significantly over the nesting season (Table 4).

**Table 4:** Significant results of least-squares linear regressions comparing plasma electrolytes, minerals, and proteins in nesting leatherback sea turtles (*Dermochelys coriacea*) to date of nesting season. Equations for the regression lines are provided, in addition to the confidence intervals (CI) of the slopes of the regression lines and the y-intercept.

Analyte	Regression equation	Slope 95% CI	Intercept 95% CI	*r*^*2*^	*P*	N
Potassium	y = –0.008x + 332.85	(–0.01, –0.002)	(69.90, 595.80)	0.22	0.016	26
Albumin:globulin	y = –0.002x + 88.86	(–0.003, –0.001)	(40.84, 136.87)	0.38	<0.001	26
Albumin	y = –0.05x + 2234.03	(–0.08, –0.02)	(834.85, 3633.21)	0.31	0.003	26

## Discussion

This study provides a comprehensive analysis of the effect of hemolysis on various blood chemistry analytes in nesting leatherback sea turtles from Florida and reports reference intervals for these analytes using non-hemolyzed plasma. Hemolysis effects were substantial despite being investigated using dry slide chemistry, which is generally less affected by interferents such as hemolysis and lipemia than wet chemistry [33]. Conversely, the magnitude of interference would be presumptively more pronounced with wet chemistry. The degree of hemolysis interference in leatherback turtles suggests unique species-specific RBC chemical components in this taxon in comparison to previously studied species. In light of this, moderately (2+) to severely hemolyzed (3+) plasma samples should be excluded from chemistry analysis for the investigated analytes, but such samples may be suitable for other analyses (e.g., toxicology, nutritional, molecular).

Visual evaluation of serum or plasma color is an important aspect in specimen accession in any diagnostic laboratory; severe hemolysis may lead to rejection of a sample [2]. Hemolysis is considered an important economic factor in human diagnostic laboratories, since it is the most frequent reason for sample rejection and because of the costs of resampling [34]. Although the economic factor may not be as relevant for veterinary diagnostic laboratories, resampling blood may not be an option after sample collection in the field for sea turtles or other wildlife species.

Plasma color in sea turtles is typically pale yellow/straw-colored and may be bright yellow in some nesting sea turtles, with reported discolorations including hemolysis, lipemia, and green (biliverdinemia) or brown color [1]. Semiquantitative assessment of the degree of hemolysis by automated chemistry analysis is considered superior over visual assessment in human laboratory medicine [3]. However, semiquantitative assessment is not often performed in veterinary diagnostic laboratories. Visual assessment of hemolysis is often the only available methodology in veterinary laboratories, but even that information may not be reported or considered in interpretation of data. Table 5 provides an overview of common field or sample handling challenges and recommendations for avoidance of hemolysis during field blood sampling, handling, and processing. After excluding all potential causes of hemolysis in a given sample, the conclusion of possible *in vivo* hemolysis can be made when interpreted in the context of relevant clinical and other laboratory findings.

**Table 5:** Challenging field or sample handling conditions potentially resulting in hemolysis and recommendations for avoidance.

Potential cause for hemolysis	Solutions	Comments
Difficult blood withdrawal	Attempt sampling from another site; *never* more than 3 attempts from 1 site Choose small size syringes	Ensure proper cleaning and disinfection (*e*.*g*., betadine + alcohol preferred) as for any sampling site Use of syringe preferred; vacutainers may cause vessels to collapse Choose appropriate needle size for size of the animal
Direct contact of blood tube with wet ice	Insulate blood tubes from direct contact with ice (e.g., bubble-wrap in between ice and blood tubes)	*Never* put whole blood in contact with dry ice as the sample will hemolyze
Exposure of blood tube to warm temperatures	Place blood tube in a cooler with ice packs; *at least* place in cold area away from sunlight	*Always* avoid exposure to heat/direct sunlight
Delays in sample processing	Plan logistics ahead of time; investigate field conditions beforehand; ensure sample processing at *earliest* possibility	*Always* ensure appropriate cooling during all steps of sample storage
Blood anticoagulation into ethylenediaminetetraacetic acid (EDTA/purple tops)	*Always* choose heparinized blood tubes for any chelonian species	EDTA is known to lyse reptile red blood cells
Too much inversion (*e*.*g*., too vigorous, too frequent)	Slow inversion of tube up to 10 times to mix whole blood and anticoagulant	Ensure proper training of personnel involved in sample handling and processing

Understanding hemoglobin concentrations can be a valuable component of the hemogram evaluation in any species. Hemoglobin measurements have traditionally been based on the cyanmethemoglobin technique which utilizes toxic reagents and also requires a centrifugation step of the blood-cyanmethemoglobin mixture to remove free nuclei before measurement of optical density [5]. Hemoglobin analysis by the HemoCue^®^ Hb 201^+^ photometer was previously validated with frozen-thawed and fresh whole blood in birds, and fresh whole blood in hawksbill sea turtles (*Eretmochelys imbricata*) [21–23]. Given that the comparison of frozen-thawed whole blood analyzed with and without centrifugation did not identify a statistical difference in our study or in birds [23], this finding indicates that the centrifugation step is not necessary when the hemoglobinometer is used with frozen-thawed blood from species with nucleated RBC. The positive and proportional correlation of PCV with hemoglobin concentration confirmed that in clinically normal leatherback sea turtles, similar to mammals and other non-mammalian species, the hemoglobin concentration is about 3 times the PCV (PCV x 3.25) using the SI unit (g/L) or one third of the PCV (PCV x 0.325) using the conventional unit (g/dL) in healthy animals. Furthermore, if only a minimal amount of blood can be obtained (e.g., in hatchlings), the minimal amount of 20 μl needed for hemoglobin analysis can be useful in estimating the PCV, and the remaining blood be centrifuged for higher plasma yield (K. Fleming, unpublished data). This information can also be used as an additional end point when interpreting blood data from clinically normal leatherback sea turtles in future studies. Furthermore, the hemoglobinometer is cost-effective and easy to operate, which makes hemoglobin analysis a suitable additional endpoint for sea turtle health assessment studies.

Electrolytes were altered by hemolysis to a degree that could affect proper data interpretation. This applies especially to potassium, suggesting that leatherback sea turtles and presumptively other sea turtle species, are among those species with high intra-erythrocytic potassium concentrations and/or have differences in erythrocyte electrolyte membrane transporters (e.g., Na-K-ATPase) [5]. Pseudohyperkalemia in mammals and humans can result from hemolysis, prolonged contact of erythrocytes with serum or plasma, thrombocytosis from potassium release of platelets after clotting for serum harvest, leukocytosis, or accidental use of EDTA plasma [3,5]. In our study, the use of frozen-thawed blood resulted in release of intracytoplasmic components of all blood cells, including RBC, white blood cells, and thrombocytes. To the authors’ knowledge, intracellular potassium concentrations of non-mammalian white blood cells (WBC) or thrombocytes are unknown. However, relative to WBC and thrombocyte numbers in healthy turtles, RBC numbers are by magnitudes higher (e.g. 65 times based on Deem *et al*., 2006 [12]). Therefore, contributions of potassium from lysed WBC and thrombocytes, when present in expected numbers in a clinically normal sea turtle are likely negligible, even if they contain small amounts. In addition, study turtles were considered clinically healthy and none of the study animals had concerns for leukocytosis or thrombocytosis (data not included). Since heparinized plasma but not serum is used for chemistry analysis in sea turtles for various reasons [1], the concern for potassium release from thrombocytes does not apply to routine plasma samples. True hyperkalemia is an important laboratory interpretation as potassium is an essential electrolyte linked with regulation of blood gases, muscle tissue, kidney function, and hormonal regulation [5]. Therefore, spurious potassium increases that fall outside the reference interval can affect clinical diagnosis and treatment decisions on an individual animal level, since cardiac effects (e.g., arrhythmia) of true hyperkalemia can be a serious clinical concern [35]. Concern for hyperkalemia due to exertion is one of the reasons why health monitoring for leatherback sea turtles during field responses or studies is important [35]. Correction formulas for estimation of actual potassium concentration in a sample affected by hemolysis have been developed for human laboratory medicine but are not recommended since hemolysis results in higher potassium concentrations than can be corrected by calculation [36]. This is consistent with our attempt to develop a correction formula for potassium in leatherback sea turtles, which did not produce a useful formula (Table 3). Although sodium and chloride were not affected to the degree of potassium, their spurious increases were significant, with observed concentrations outside the reference interval. This could affect laboratory data interpretation associated with hydration status and functional assessment of kidneys, gastrointestinal tract, and salt glands [1,5].

Minerals were variably affected by hemolysis, but overall less than electrolytes. While calcium was not significantly increased, all minerals including calcium were observed outside their established reference intervals, and significant increases were observed in phosphorus, calcium:phosphorus ratio, and magnesium. This has the potential to alter interpretation of mineral homeostasis, vitamin D metabolism, and plasma biochemical evaluation of bones, muscles, and kidneys. Although the calcium:phosphorus ratio is not used in marine turtles as in terrestrial reptiles in the laboratory evaluation of renal function, it can provide useful information in sea turtles regarding diet, tissue growth, and life-stage class differences [1]. Hence, accurate interpretative information gets lost in the presence of sample hemolysis.

Plasma protein fractions are important chemistry analytes in reptiles, since they enable evaluation of overall health and nutrition, inflammation, and immune and various organ functions such as gastrointestinal tract, liver, and kidneys, when interpreted in context of other plasma chemistry analytes [1,5]. All protein fractions except for pre-albumin were affected by moderate hemolysis in leatherback sea turtles. Since total protein measurement by biuret method and plasma protein electrophoresis is the gold standard to obtain accurate plasma albumin in reptiles [37], falsely increased albumin may mask hypoalbuminemia in leatherbacks. The effect on globulins was expected, since globin chains of hemoglobin migrate into the alpha- or beta-globulin regions in various species, specifically beta in dogs, gamma in birds, beta_2_ in green iguanas (*Iguana iguana*), and alpha_1_ and beta in pond sliders (*Trachemys scripta*) [5,7,38]. Interestingly, hemolysis resulted in distinct peaks in the alpha_2_-globulin fraction in nesting leatherback sea turtles in this study (Fig 4), expanding on species-specific differences that may be due to size and/or migration pattern of globin chains. This finding is important since this protein fraction typically increases with inflammatory responses and would alter interpretation of electrophoretic patterns of plasma proteins.

Our conversion equations could theoretically be used by leatherback researchers and clinicians to estimate non-hemolyzed plasma data using those from hemolyzed plasma samples. In practice, however, the relatively poor correlations between hemolyzed and non-hemolyzed values raise concern regarding their utility. Most of the equations, except for the calcium:phosphorus ratio (*r*^2^ = 0.951), were weakly to moderately correlated at best (*r*^*2*^ < 0.745 in all other blood analytes). The lack of any significant correlations between values of phosphorous, pre-albumin, alpha_1_-globulins, and potassium is especially concerning. This absence of model fit makes predicting non-hemolyzed values from hemolyzed ones nearly impossible and raises concern regarding the interpretation of these values from hemolyzed samples. Further research is needed to investigate red blood cell chemistry of leatherbacks and conversion factors accounting for hemolyzed plasma samples.

Blood analyte data of leatherback sea turtles of various life-stage classes and from different geographical locations have been documented [14–15,17,35], including chemistry data from nesting leatherbacks from Equatorial Guinea, Gabon, and French Guiana [12,16,39], and protein electrophoresis data from nesting leatherbacks from Florida and St. Croix, U.S. Virgin Islands [18–19]. The established reference intervals in this study are representative for female nesting leatherbacks over a two-month period covering ~84% of the total nesting activity in this location, reflecting overall blood analyte data for this life-stage class from Florida, similar to those reported by Deem *et al*. (2006) [12]. Slight variations are overt in comparing our results to previous reports. These variations may be attributed to differences in sample handling, processing, and analytical methodologies, and possibly different times of sample collection during months of the nesting season over time [16,18–20,39]. The lower trending potassium, albumin, and A:G ratio across the nesting season in our study are consistent with other reports of nesting leatherbacks and hawksbills in which changes in these analytes were attributed to reduced food intake [16,18–19,40]. Potential additional trends may not have been detected in our study given the relatively low sample size. Further studies are warranted (e.g., metabolomics) to investigate blood analyte changes in nesting leatherback sea turtles from Florida to further the understanding of the dynamics of energy metabolism and possible fasting across nesting season in this species.

Packed cell volume in nesting leatherbacks from Florida in this study is similar to nesting leatherbacks from Gabon, Equatorial Guinea, and Papua New Guinea [12,15–16], which is collectively lower compared to foraging leatherbacks in the northwest Atlantic [17] and eastern Pacific [15]. Lower trending PCV is common in leatherbacks during nesting season [1,16,18,20,39]. Hemoglobin quantification has not been reported in leatherback sea turtles to date, but results are much higher than in loggerheads (*Caretta caretta*), green turtles (*Chelonia mydas*) and hawksbill sea turtles ([21,41–42]; Perrault, unpublished data). Although methodology differences among studies may factor into the observed difference to some degree, the comparatively higher hemoglobin in addition to the observation of higher PCV in leatherback sea turtles compared to other chelonids suggest higher blood oxygen-carrying capacity in this species. This consideration is supported by differences in diving physiology (e.g., longer and deeper dives), metabolic rates, and migration behavior compared to other sea turtle species [20,43–44].

### Conclusions

Non-hemolyzed plasma samples provide the best quality for chemistry analysis of electrolytes, minerals, and proteins in leatherback sea turtles. Our results show that moderate hemolysis (2+) can alter results to a degree that data can fall outside of the expected reference intervals. Such artifactual derangements could lead to erroneous clinical decisions (e.g., impacting decisions on treatment for an individual animal), or lead to misinterpretation of population-level data in sea turtle health studies (e.g., long-term database and/or multi-year studies). Consequently, all efforts should be undertaken to avoid sample hemolysis along all steps of sample collection, handling, and processing. If sample hemolysis is present, it is important to characterize the degree of hemolysis based on visual evaluation or, if available, based on an automated technique for quantifying the hemolysis index. Through the use of the handheld hemoglobinometer, it was possible to understand hemoglobin concentration-driven changes on plasma chemistry analytes, and to identify a proportional relationship of hemoglobin and PCV. These findings may be useful in interpretation of erythrogram data, and when planning studies where only a very limited blood volume can be collected. In summary, this study provides information that will be useful for planning field blood sample handling and processing in order to ensure optimal sample quality from leatherback sea turtles. This will ultimately result in high quality data for use in conservation-related projects.
